# Effect of dietary aloe vera on growth and lipid peroxidation indices in rainbow trout (*Oncorhynchus mykiss*)

**Published:** 2015-03-15

**Authors:** Ghazale Golestan, Amir Parviz Salati, Saeed Keyvanshokooh, Mohammad Zakeri, Hossein Moradian

**Affiliations:** 1 *Department of Fisheries, Faculty of Marine Natural resources, Khorramshahr University of Marine Science and Technology, Khorramshahr, Iran;*; 2 * Coldwater Fishes Genetic and Breeding Research Center, Iranian Fisheries Research Organization, Yasuj, Iran.*

**Keywords:** Aloe vera, Lipid peroxidation, Malondialdehyde, *Oncorhynchus mykiss*

## Abstract

Aloe vera has been used worldwide in pharmaceutical, food and cosmetic industries due to the plethora of biological activities of its constituents. This study was done to evaluate the effects of dietary aloe vera on growth and lipid peroxidation in rainbow trout* (Oncorhynchus mykiss)*. A total number of 480 *O. mykiss* (mean weight 9.50 ± 0.85 g) were randomized into four experimental groups including one control and three experimental groups that aloe vera was incorporated in their diet at 0.5, 1.0 and 2.0 g kg^-1^. Trial was done for eight weeks. Then biometry and blood sampling were done. Plasma malondialdehyde, ferric reducing ability of plasma and growth index were estimated at the end of study. The results showed that aloe vera extract did not affect growth indices. Malondialdehyde was increased in the experimental group compared to the control group but ferric reducing ability of plasma showed a decrease in experimental groups (*p *< 0.05) compared to the control group. Our findings showed that dietary aloe vera have adverse effects on antioxidant defense system in *O. mykiss*.

## Introduction

*Aloe barbadensis* Miller (Aloe vera) is a tropical or subtropical plant characterized by lance-shaped leaves with jagged edges and sharp points that belongs to Liliaceae or Aloeaceae family.^1^ The leaves are 30 to 50 cm long and 10 cm broad at the base. Color is pea-green, white spotted while young, and has bright yellow tubular flowers, 25 to 35 cm in length arranged in a slender loose spike. It contains a colorless mucilaginous gel called aloe vera gel.^2^ Several biological effects of aloe vera including immuno-stimulation, increasing growth performance, improvement of wound healing in fish have been reported.^3^^- ^^5^

Reactive oxygen species (ROS) are continually produced in animals during normal aerobic metabolism that can damage most cellular components leading to cell death.^6^ Fish like other vertebrates possess an antioxidant defense system to reduce the negative effects of ROS. Main enzymes involved in this system are superoxide dismutase (SOD), catalase (CAT), glutathione peroxidase (GSH-Px), glutathione reductase (GR), glucose-6-phosphate dehydro-genase (G6PD) and glutathione S-transferase (GST).^7^ Fish like all aerobic organisms are susceptible to ROS,^8^ however, most of data about oxidative defense in fish are focused on natural and anthropogenic toxicological aspects especially environmental factors and xenobiotics.^9^ In different studies effect of different diets on fish antioxidant defense had been assayed.^10^^,^^11^ *In vivo* antioxidant capacity of organic extracts of aloe vera leaf had been shown.^12^^,^^13^ Therefore, this study was performed to evaluate effects of aloe vera on growth and lipid peroxidation in rainbow trout, *Oncorhynchus mykiss*.

## Materials and Methods

**Diets. **Basal diet based on formulation of 48.25% protein, 18.86% lipid, 8.23% carbohydrates and 9.36% moisture was prepared (Table 1). Experimental diets were produced adding appropriate amount of aloe vera gel (Barij Essence Pharmaceutical Co., Kashan, Iran) to make experimental diets as follows: 0.5, 1.0 and 2.0 g kg^-1^ meal, respectively, in groups 1, 2 and 3. Feeds were dried in the oven separately and then were sent to oiling process to be covered with fish oil. In control group aloe vera was not added to basal diet. The composition of the diets is shown in Table 1.

**Table 1:** Proximate composition of the basal diets fed to *Oncorhynchus mykiss*

**Diet ingredient**	**Amount**
**Protein (%)**	48.25
**Fat (%)**	18.86
**Moisture (%)**	9.36
**Ash (%)**	12.25
**Carbohydrate (%)**	8.23
**Energy (KJ kg** ^-1^ **)**	2025

**Experimental design. **A total number of 480 rainbow trout were acclimatized for one week in 300 L aquaria. After this period fish (average body weight of 9.50 ± 0.85 g; average length 9.46 ± 1.10 cm) were randomly divided into 12 aquariums (300 L) each containing 40 fish, arranged in a flow-through system. Each treatment was done in triplicate. The fish were kept for eight weeks in controlled condition (Temperature 11.30 ± 0.80 ˚C, pH = 7.92 ± 0.68 and dissolved oxygen = 7.80 ± 0.48 mg L^-1^) under natural photoperiod and fed at 3.00% body weight per day spread across the feeding times (08:00 and 15:00). Environmental parameters were controlled daily. The survival was controlled daily. The feeding ration was corrected every two weeks following 24 hr starvation and batch weighing. At the end of experiment all of fish in each tank were individually weighed 24 hr after the last feeding. 

**Growth Performance. **To define the growth indices of the *O. mykiss*, biometry was done in all groups once every two weeks during the experiment. After eight weeks, all of the fish in experimental groups weighed by digital scale (bearing: 0.01 mg). Specific growth rate (SGR), feed conversion ratio (FCR) and condition factor (CF) were calculated using standard formula.^14^

*Weight Gain (WG) = W*_f_ *- W*_i_

*Feed conversion rate (FCR) = Food intake/Weight gain*

*Condition factor (CF) = Wet body weight × 100 / (Total body length)* ^3^

*Specific growth rate (SGR) = 100 × (Ln W*_f_ *– Ln W*_i_ *)/Days*

where, *Ln* is natural logarithm, *W*_f_ is mean final weight (g) and *W*_i_ is mean initial weight (g).

**Biochemical analysis. **The blood samples were taken through the severing the caudal peduncle and stored in heparinized tubes. For biochemical assays, blood samples were centrifuged at 5000 *g* for 5 min (Model D7200; Hettich, Tuttlingen, Germany) at room temperature immediately then plasma was separated, pooled (4 samples per pool) and stored at – 80 ˚C until analysis. Total protein and albumin were assayed by biuret, and Bromocresol green binding method and globulin were calculated by distracting albumin from total protein.

The total amount of plasma lipid peroxidation was indicated by the content of malondialdehyde (MDA) as described by Buege and Aust.^15^ Briefly, one volume of plasma was mixed with two volumes of a stock solution of 15.00% w/v trichloroacetic acid, 0.375% w/v thiobarbituric acid and 0.25 molhydrochloric acid thoroughly. The solution was heated for 15 min in boiling water bath. After cooling, the precipitate was removed by centrifugation at 1000 *g* for 10 min. The absorbance of the clear supernatant was determined at 535 nm.

The FRAP reagent included 10mM 2,4,6-Tri (2-pyridyl)1,3,5-triazine (TPTZ) solution in 40mM HCl, 20 mM FeCl solution and 0.3 M acetate buffer (pH=3.6) in proportions of 1:1:10 (v/v). 50 l of each dilutedethanolic extracts were mixed with 3 mL of freshly prepared FRAP reagent and the reaction mixtures incubated at 37 ˚C for 30 min. Absorbance at 593 nm was determined against distilled water blank. FRAP values were expressed as µmol of Fe in each mL of plasma.^16^

**Statistical analysis. **All data are presented as mean ± SD. The SPSS (Version 13; SPSS Inc., Chicago, USA) was used for analysis. Data were analyzed by one-way ANOVA followed by Tukey’s multiple comparisons test. A *p*-value less than 0.05 was determined as a significant difference.

## Results

No mortalities were recorded during the trial. As seen in Table 2 at the end of feeding period, dietary aloe vera did not affect growth indices in *O. mykiss* and a significant change was not recorded between control and experimental groups (*p *> 0.05), (Table 2). Feed conversion rate was 0.01 ± 0.43 in control group and was not affected by dietary aloe vera as a significant change was not recorded in experimental groups compared to control group (*p *> 0.05). Changes in body condition factor showed a similar result as a significant change was not seen in experimental groups in comparison to control.

Malondialdehyde levels showed variation between experimental groups but was significantly higher in fish fed 2.0 g kg^-1^ aloe vera (*p *< 0.05). Plasma MDA content was higher in fish fed with 2 g kg^-1^ aloe vera compared with those fed with 1.0 g kg^-1 ^aloe vera (*p *< 0.05), but both of them did not show significant changes compared with group that fed with the lowest dose of aloe vera. Ferric reducing antioxidant was only affected in fish fed with 0.5 g kg^-1 ^aloe vera compared with control as it was decreased significantly (*p* < 0.05) but in other experimental groups did not show a significant change compared with control group (Table 3). There were no differences between the experimental groups.

**Table 2:** Growth performance in *Oncorhynchus mykiss* fed with control diet, 0.5, 1.0 and 2.0 g kg^-1 ^aloe vera for eight weeks. Data are presented as mean ± SD.

**Parameters**	**Control**	**Aloe vera concentration in diet (g kg** ^-1^ **)**		
		**0.5**	**1.0**	**2.0**
**Initial body weight (g)**	0.31 ± 9.53	0.54 ± 9.76	0.10 ± 9.15	0.31 ± 9.47
**Final body weight (g)**	0.95 ± 53.63	1.49 ± 60.82	4.38 ± 59.03	3.54 ± 54.02
**Weight gain ** **(g)**	1.26 ± 44.09	1.089 ± 51.06	4.49 ± 49.88	3.02 ± 44.88
**Relative growth rate (%)**	0.01 ± 0.44	0.01 ± 0. 50	0.04 ± 0.49	0.01 ± 0.44
**Specific** ** growth rate ** **(%)**	0.04 ± 6.30	0.06 ± 6.54	0.14 ± 6.49	0.09 ± 6.31
**Feed conversion ratio (%)**	0.01 ± 0.43	0.05 ± 0.42	0.01 ± 0.44	0.01 ± 0.43
**Body condition**	0.04 ± 1.47	0.05 ± 1.49	0.05 ± 1.49	0.04 ± 1.54

**Table 3:** Effect of dietary aloe vera on plasma malondialdehyde concentration and ferric reducing ability of *Oncorhynchus mykiss*. Data are presented as mean ± SD.

**Parameters**	**Control**	**Aloe vera concentration in diet (g kg** ^-1^ **)**		
		**0.5**	**1.0**	**2.0**
**Ferric reducing ability** ** (μmol** **L**^-1^**)**	747.83 ± 96.83^a^	878.80 ± 103.61^b^	691.70 ± 91.97^ab^	975.01 ± 81.15^ab^
**Malondialdehyde** ** concentration ** **(** **μmol** **mL**^-1^**)**	66.83 ± 3.38^a^	31.65 ± 5.28^ab^	52.56 ± 7.63^a^	25.95 ± 2.81^b^

ab Different superscripts letters in each row indicate significant difference (*p* < 0.05).

## Discussion

Although effects of aloe vera on some animals had been studied, there is lack of information about its effect on the antioxidant system of *O. mykiss* and the other aquatic species. In this study aloe vera did not affect growth parameters in experimental groups. In *Amphiophus labiatus* fed with different levels of aloe vera similar results was recorded as a dose dependent response changes in growth parameters was not seen.^17^ Farahi *et al.* showed that aloe vera (10 g kg^-1 ^of diet) and *Melissa officinalis* had no effect on growth performance in *O. mykiss*.^18^ Heidarieh *et al.* demonstrated that high doses of dietary aloe vera had a positive effect on rainbow trout growth performance.^5^ In Gold fish, *Carassius auratus* maximum specific growth rate and weight gain was recorded in fish fed with diet containing 0.5 g kg^-1 ^aloe vera.^19^ Controversial reports on growth promoting effects of aloe vera that also reported for other herbal additives may be caused by different amount of additive, composition of basal diet, management and husbandry conditions.^20^^,^^21^

Antioxidant effects of plants containing compounds such as flavonoids, phenolic compounds, ascorbic acid and tocopherol have been reported.^22^ The aloe had polysaccharides,^23^ antioxidant enzymes,^24^ Phenolic compounds and aloesin derivatives,^25^ that could be related to effectiveness of aloe as an antioxidant in some reports. In contrast to clinical reports of the useful activity with aloe gel,^26^ there were also a few cautionary accounts on its harmful effect.^27^

Malondialdehyde levels show the degree of lipid peroxidation directly and damage cell indirectly.^28^ The lowest level of MDA was observed in the control group and increased in experimental groups. Kamel Mohamed found that treatment with aloe vera caused an increase in antioxidant activity in diabetic rats.^29^ It also significantly reduced the lipid peroxidation and MDA production. In some studies beneficial effects of the aloe vera on antioxidant defense has been reported. Use of aloe vera juice enhanced the levels of antioxidant enzymes in mouse.^30^ Anilakumar *et al.* showed that some constituents of aloe vera gel extract such as Barbalvyyn, glucomannan, asmanan, minerals, flavonoids and tannic acid may be responsible for reducing oxidative stress and induced toxicity.^12^ Farahi *et al.* found that phenolic compounds (flavonoids, proanthocyanidin) have strong antioxidant activity for the removal of free radicals and oxidative reactions are completed.^18^ The FRAP shows extracts reducing power and its ability to reduce Fe^3+^ to Fe^2+^. It had been reported that reducing power of aloe vera like other medicinal plants is related to phenolic constituents.^11^ Our findings suggested that in *O. mykiss*, phenolic constituents of aloe vera did not enhance antioxidant activity, even reduced it. Decrease in FRAP may be caused by inhibition of activity of superoxide dismutase, catalase and glutathione peroxidase in *O. mykiss* following dietary administration of aloe vera.^31^

Our results showed that dietary administration of aloe vera in *O. mykiss* t did not have positive effect on growth performance but also adversely affected lipid peroxidation that resulted to increase in MDA.
